# Anemia in Patients With Resistance to Thyroid Hormone *α*: A Role for Thyroid Hormone Receptor *α* in Human Erythropoiesis

**DOI:** 10.1210/jc.2017-00840

**Published:** 2017-07-11

**Authors:** Anja L. M. van Gucht, Marcel E. Meima, Carla Moran, Maura Agostini, Anna Tylki-Szymanska, Malgorzata Krajewska, Walasek, Krystyna Chrzanowska, Alexandra Efthymiadou, Dionisios Chrysis, Korcan Demir, W. Edward Visser, Theo J. Visser, Krishna Chatterjee, Thamar B. van Dijk, Robin P. Peeters

**Affiliations:** 1 Department of Internal Medicine, Erasmus University Medical Center, 3000 Rotterdam, The Netherlands; 2 Wellcome–Medical Research Council Institute of Metabolic Science, University of Cambridge, CB2 0QQ Cambridge, United Kingdom; 3 The Children’s Memorial Health Institute, 04-730 Warsaw, Poland; 4 Department of Pediatrics, Division of Endocrinology, University of Patras Medical School, 25002 Patras, Greece; 5 Division of Pediatric Endocrinology, Faculty of Medicine, Dokuz Eylül University, 35100 Izmir, Turkey; 6 Department of Cell Biology, Erasmus University Medical Center, 3000 Rotterdam, The Netherlands

## Abstract

**Context:**

Patients with resistance to thyroid hormone (TH) *α* (RTH*α*) are characterized by growth retardation, macrocephaly, constipation, and abnormal thyroid function tests. In addition, almost all RTH*α* patients have mild anemia, the pathogenesis of which is unknown. Animal studies suggest an important role for TH and TH receptor (TR)*α* in erythropoiesis.

**Objective:**

To investigate whether a defect in TR*α* affects the maturation of red blood cells in RTH*α* patients.

**Design, Setting, and Patients:**

Cultures of primary human erythroid progenitor cells (HEPs), from peripheral blood of RTH*α* patients (n = 11) harboring different inactivating mutations in TR*α* (P398R, F397fs406X, C392X, R384H, A382fs388X, A263V, A263S), were compared with healthy controls (n = 11). During differentiation, erythroid cells become smaller, accumulate hemoglobin, and express different cell surface markers. We assessed cell number and cell size, and used cell staining and fluorescence-activated cell sorter analysis to monitor maturation at different time points.

**Results:**

After ∼14 days of *ex vivo* expansion, both control and patient-derived progenitors differentiated spontaneously. However, RTH*α*-derived cells differentiated more slowly. During spontaneous differentiation, RTH*α*-derived HEPs were larger, more positive for c-Kit (a proliferation marker), and less positive for glycophorin A (a differentiation marker). The degree of abnormal spontaneous maturation of RTH*α*-derived progenitors did not correlate with severity of underlying TR*α* defect. Both control and RTH*α*-derived progenitors responded similarly when differentiation was induced. T3 exposure accelerated differentiation of both control- and RTH*α* patient–derived HEPs.

**Conclusions:**

Inactivating mutations in human TR*α* affect the balance between proliferation and differentiation of progenitor cells during erythropoiesis, which may contribute to the mild anemia seen in most RTH*α* patients.

## Teaser

In this study, we found that mutations in human TR*α* affect the balance between proliferation and differentiation of erythroid progenitor cells, which may contribute to anemia in most RTH*α* patients.

Erythropoiesis is the process that involves the maturation of hematopoietic progenitor cells to differentiated red blood cells (erythrocytes). Erythrocytes are of fundamental importance for all vertebrates because they provide cells with oxygen in exchange for carbon dioxide (1, 2). The site of erythropoiesis changes throughout human development. During early embryonic development, erythropoiesis occurs in the yolk sac. By the third to fourth month of gestation, this primitive function is taken over by the liver. From the seventh month of gestation onward and throughout adulthood, the bone marrow is the predominant erythropoietic organ (3–6).

The production of sufficient numbers of mature red blood cells requires a fine balance between proliferation and differentiation of progenitor cells. The cytokine erythropoietin (Epo) plays a key role in this process, along with other growth factors, such as interleukin 3, stem cell factor (SCF), and insulinlike growth factor I (2, 7, 8). In addition, thyroid hormone (TH) is also important for erythropoiesis. Patients with hypothyroidism frequently have anemia (9), and mice with congenital primary hypothyroidism are also anemic (10). The action of TH is mediated via binding of the active ligand (T3) to nuclear TH receptors (TRs), TR*α* and TR*β*. TRs are ligand-inducible transcription factors that regulate target gene expression by binding to TH response elements in promoters of T3-responsive genes (11–13).

Interestingly, the presence of v-ErbA, an oncogenic homolog of TR*α*, disturbs the balance between proliferation and differentiation of immature avian erythrocytes during erythropoiesis, contributing to fatal erythroleukemia (14–16). Additional evidence for the involvement of T3 and TR*α* in erythropoiesis is provided by observations in mice lacking TR*α* (TR*α*^−/−^), showing compromised fetal and adult erythropoiesis with fewer erythroid progenitors in TR*α*^−/−^ fetal livers and impaired transit of TR*α*^−/−^ erythroblasts through further stages of maturation (17). Other studies with TR*α* knockout mice, displaying defective spleen erythropoiesis, confirm that T3 via TR*α* stimulates late steps of erythroid development (18).

In 2012, the first patients with resistance to TH*α* (RTH*α*) due to inactivating mutations in *THRA* were discovered. All patients identified since then have monoallelic mutations in the ligand-binding domain of TR*α*. The phenotype of RTH*α* patients is characterized by growth restriction, varying degrees of neurodevelopmental retardation, macrocephaly, constipation, and abnormal thyroid function tests (low/low-normal FT4 and high/high-normal T3 levels with a normal thyrotropin) (19–28). In addition, a mild, usually normochromic and normocytic anemia is a virtually universal finding in RTH*α* patients. However, in three cases the mean corpuscular volume was raised (20, 22, 27).

Given the observations that most RTH*α* patients have anemia and that aberrant TR*α* signaling affects erythropoiesis in animal models, we hypothesized that mutations in TR*α* affect the balance between proliferation and differentiation in the later stages of human erythropoiesis.

## Patients, Materials, and Methods

### Cells and cell culture

Peripheral blood (5 to 10 mL) was obtained by venesection and collected into heparin or EDTA tubes from 11 RTH*α* patients, who have been described previously (21–23, 25, 28), and 11 healthy donors (n = 3 related and n = 8 nonrelated). The study was approved by the Medical Ethics Committee of the Erasmus Medical Center. Written informed consent was obtained from all subjects and/or their parents. Mononuclear cells were purified from peripheral blood by density gradient centrifugation using Ficoll (Axis-Schield, Oslo, Norway).

Human erythroid progenitor cells (HEPs) were expanded in StemSpan^TM^ Serum-Free Expansion Medium (Stem Cell Technologies, Grenoble, France) supplemented with lipids (40 μg/mL cholesterol-rich lipid mix; Sigma-Aldrich, St. Louis, MO), penicillin-streptomycin (1:100; Lonza, Basel, Switzerland), recombinant human Epo (2 U/mL; Janssen-Cilag, Baar, Switzerland), recombinant human SCF (100 ng/mL; R&D Systems, Minneapolis, MN), human interleukin 3 (1 ng/mL; R&D Systems), human insulinlike growth factor I (40 ng/mL; R&D Systems), and dexamethasone (Dex; 1 *μ*M; Sigma-Aldrich) (29, 30). After 4 to 5 days, HEPs were purified by density purification (Percoll; GE Health Care, Little Chalfont, UK) and further expanded in StemSpan containing Epo, SCF, and Dex.

After sufficient expansion (10 to 15 days), 10 nM T3 (Sigma-Aldrich) was added to part of the HEPs, cultured under proliferation conditions. To induce differentiation, HEPs were washed three times with phosphate-buffered saline (Lonza) and switched to StemSpan medium containing Epo (10 U/mL), human serum (3%; Sigma-Aldrich), and iron-saturated transferrin (1:100; Scipac, Kent, UK).

HEPs were maintained at 1 to 1.5 × 10^6^ cells/mL during proliferation and at 1.5 to 2 × 10^6^ cells/mL during differentiation by daily partial medium changes. To confirm that these cells are a good TR*α*-expressing model, RNA was extracted using TRI Reagent (Sigma-Aldrich), and deep sequencing was performed [using the Illumina Hiseq2500 platform (single read 43 bp)] in a subset of control HEPs during late proliferation (Supplemental Fig. 1).

### Cell count and morphology

The number of HEPs was daily monitored by cell count (CASY^®^ INNOVATIS TTC Cell Counter; Omni Life Science, Raynham, MA). To analyze cell morphology at various stages of proliferation and differentiation, 10^5^ HEPs were centrifuged onto glass slides and stained with Benzidine (Sigma-Aldrich) to detect hemoglobin-expressing cells, and with Diff-Quick staining (Medion Diagnostics, Miami, FL). Images were made using an Olympus BX40 microscope (×40 objective, NA 0.65; Olympus, America, Valley, PA) equipped with an Olympus DP50 charge-coupled device camera (Olympus, America) and Viewfinder lite 1.0 acquisition software (Better Light, San Carlos, CA). The images were processed using Adobe Photoshop SC6 (Adobe, San Jose, CA).

### Flow cytometry

HEPs (3 × 10^5^ cells) were briefly centrifuged, after which the pellet was resuspended in phosphate-buffered saline supplemented with 0.1% bovine serum albumin and 1 mM EDTA [fluorescence-activated cell sorter (FACS) buffer] and stained for 30 minutes at room temperature with fluorescently labeled antibodies against the following cell surface markers (all BD Pharmingen antibodies, San Diego, CA): CD117 (stem cell factor receptor c-Kit, dilution 1:100), CD71 (transferrin receptor, dilution 1:100), and CD235a [glycophorin A (GPA), dilution 1:1000] in a final volume of 100 μl. To exclude nonviable cells, 7-aminoactinomycin D (A1310; Thermo Fisher Scientific, Waltham, MA; dilution 1:1200) was added to all samples. Fluorescence was measured on a FACS Fortessa instrument (BD Biosciences, Oxford, UK), and data were analyzed using FlowJo v.10.1 software (FlowJo, Ashland, OR).

## Results

### Genotypic and phenotypic characterization of RTH*α* patients

All 11 patients were heterozygous for a mutation in the C-terminal, ligand-binding domain of TR*α*1. In patients 5 to 9 (P5 to P9), the mutation (A263S/V) also affected TR*α*2. When studied *in vitro*, the mutant receptors showed defective hormone-dependent activation and inhibited wild-type TR*α*1 function in a dominant-negative manner when coexpressed (22, 24). The severity of mutant receptor dysfunction varied depending on the type of the mutation. Patients 1 to 3 (P1 to P3) have frameshift/premature stop, and P4 a nonsense mutation, all generating a truncated receptor with negligible T3-induced transcriptional activity and marked dominant-negative activity. In contrast, P5 to P11 have missense mutations that cause reduced T3 sensitivity.

All RTH*α* patients with a severe mutation (P1 to P4), as well as most patients with milder mutations (P5 to P7 and P9 to P11), exhibited mild anemia. There was no correlation between the severity of the mutations and hemoglobin levels or red blood cell count (Table 1). Except for P4, P9, and P11, RTH*α* patients were treated with levothyroxine (LT4).

**Table 1.** **Hematological Data of RTH*α* Patients**

Subject	Mutation	Sex	Age	LT4	Hb	Ht	RBC	MCV	MCH	Reticulocytes	Platelets	WBC
P1	F397fs406X	F	16	Yes	11.1 (12.5–16.0 g/dL)	0.34 (0.37–0.47 L/L)	3.95 (4.2–5.4 × 10^12^/L)	86.6 (78–100 fL)	28.1 (27–31 pg)	0.9 (0%–2%)	235 (150–400 × 10^9^/L)	9.07 (4.0–11.0 × 10^9^/L)
P2	F397fs406X	M	52	Yes	13.3 (13.5–18.0 g/dL)	0.38 (0.42–0.52 L/L)	4.20 (4.3–5.9 × 10^12^/L)	89.5 (78–100 fL)	31.1 (27–31 pg)	0.89 (0%–2%)	203 (150–400 × 10^9^/L)	6.47 (4.0–11.0 × 10^9^/L)
P3	A382PfsX7	F	48	Yes	12.0 (11.5–16.0 g/dL)	0.36 (0.35–0.46 L/L)	3.42 (3.8–0.5.3 × 10^12^/L)	104 (80–100 fL)	35.1 (27–32 pg)	0.67 (0.32%–2.5%)	95 (150–400 × 10^9^/L)	4.80 (4.0–11.0 × 10^9^/L)
P4	C392X	M	20	No	9.3 (12.0–16.5 g/dL)	28.4 (0.36–0.50 L/L)	3.12 (4.0–5.5 × 10^12^/L)	91.1 (80–100 fL)	29.7 (26–34 pg)		154 (150–400 × 10^9^/L)	4.6 (4.0–10.0 × 10^9^/L)
P5	A263S	M	4	Yes	11.0 (11.0–16.0 g/dL)	0.32 (0.35–0.48 L/L)	3.88 (4.0–0.6.0 × 10^12^/L)	84.4 (80–100 fL)	28.2 (28–32 pg)	0.47 (0.5%–1.5%)	255 (150–400 × 10^9^/L)	8.97 (4.5–10.5 × 10^9^/L)
P6	A263S	F	7	Yes	10.8 (11.0–16.0 g/dL)	0.32 (0.35–0.48 L/L)	3.91 (4.0–0.6.0 × 10^12^/L)	83.6 (80–100 fL)	27.6 (28–32 pg)	0.51 (0.5%–1.5%)	268 (150–400 × 10^9^/L)	4.38 (4.5–10.5 × 10^9^/L)
P7	A263S	F	31	Yes	9.6 (11.0–16.0 g/dL)	0.31 (0.35–0.48 L/L)	3.80 (4.0–0.6.0 × 10^12^/L)	82.0 (80–100 fL)	25.2 (28–32 pg)	0.43 (0.5%–1.5%)	206 (150–400 × 10^9^/L)	4.02 (4.5–10.5 × 10^9^/L)
P8	A263S	M	55	Yes	13.5 (13.5–17.5 g/dL)	0.40 (0.41–0.53 L/L)	4.34 (4.0–0.6.0 × 10^12^/L)	94.4 (80–100 fL)	31.2 (28–32 pg)	1.49 (0.5%–1.5%)	249 (150–400 × 10^9^/L)	5.18 (4.5–10.5 × 10^9^/L)
P9	A263V	M	17	No	11.9 (13.0–17.0 g/dL)	0.36 (0.37–0.49 L/L)	3.79 (4.5–0.5.3 × 10^12^/L)	94.4 (78–100 fL)	31.5 (28–32 pg)	58.9 (20–120 × 10^9^/L)	170 (150–400 × 10^9^/L)	5.9 (4.0–11.0 × 10^9^/L)
P10	R384H	F	35	Yes	10.7 (11.0–16.0 g/dL)	0.34 (0.35–0.48 L/L)	3.91 (4.0–6.0 × 10^12^/L)	86.1 (78–100 fL)	27.2 (28–32 pg)	1.60 (0.5%–1.5%)	241 (150–400 × 10^9^/L)	4.90 (4.5–10.5 × 10^9^/L)
P11	P398R	F	8	No								

Because blood samples were obtained from anonymous healthy blood donors (3 females, 8 males, age range 18 to 61 years), exact hematological data were not available. However, none of the donors had anemia because, for donation, one’s hemoglobin level is required to be within the reference range (12.5 to 17.5 g/dL for female donors, and 13.5 to 19.0 g/dL for male donors of Sanquin Blood Bank).

Abbreviations: Hb, hemoglobin; Ht, hematocrit; MCH, mean corpuscular hemoglobin; MCV, mean corpuscular volume; RBC, red blood cell; WBC, white blood cell.

### Delayed spontaneous differentiation in HEPs of RTH*α* patients

Peripheral blood mononuclear cells were isolated from RTH*α* patients and healthy controls and cultured in conditions permissive for proliferation. The population of expanding HEPs was purified by Percoll density centrifugation after 4 to 5 days, depending on the number of cycling progenitor cells at day 0. Once homogenous HEP populations were established (after 10 to 15 days), cultures were monitored daily for cell number and cell size.

**Figure 1.**
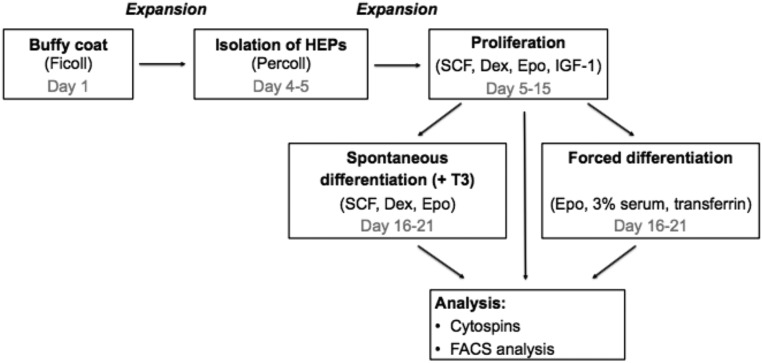
Flow chart of methods. IGF1, insulinlike growth factor 1.

**Figure 2.**
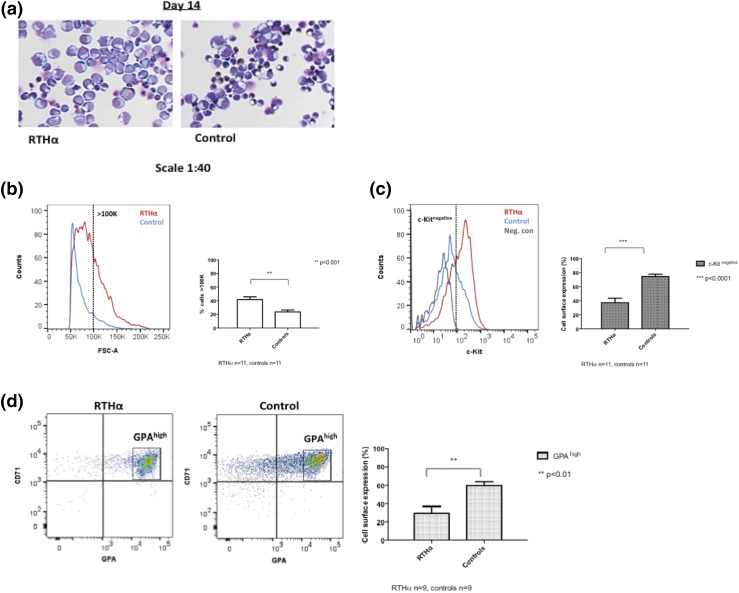
Spontaneous differentiation is delayed in HEPs from RTH*α* patients. (a) Benzidine- and Diff-Quick–stained cytospins of RTH*α*-derived (P4; C392) and control HEPs. After 2 weeks of proliferation (day 14), control HEPs start to differentiate spontaneously, illustrated by smaller and dark-stained (hemoglobin-containing) cells. In contrast, cytospin preparations from RTH*α*-derived HEPs show predominantly large, weakly stained cells that are still proliferating. (b) FACS analysis of RTH*α*-derived and control HEPs after 2 weeks of proliferation (day 14). RTH*α*-derived HEPs had a larger mean size compared with control HEPs, illustrated by the percentage of cells with a forward scatter index large than 100 K (41.7% of cells in patients vs 23.6% in controls, *P* < 0.001). (c) FACS analysis of RTH*α*-derived and control HEPs during late proliferation (culture day ∼14). RTH*α*-derived HEPs express predominantly c-Kit, a proliferation cell surface marker (37.2% of cells in patients vs 74.5% of cells in controls accumulate in the c-Kit^negative^ subgate, *P* < 0.0001). (d) In contrast, the majority of control HEPs express differentiation cell surface markers (CD71 and GPA) (29.6% of cells in patients vs 59.7% of cells in controls accumulate in the GPA^high^ subgate, *P* < 0.01).

After ∼2 weeks of proliferation, normal HEPs start to differentiate spontaneously, a process characterized by a decrease in proliferation rate, reduced cell size, cytoplasmic acidification, hemoglobin production, and nuclear condensation, followed by enucleation (31). This reduction in growth rate and cell size was delayed in the RTH*α* cells, suggesting reduced spontaneous differentiation capacity in these cells. This was further studied by Benzidine/Diff-Quick staining of cytospin preparations. Proliferating, hemoglobin-negative HEPs appear as large cells with blue cytoplasm, whereas differentiating, hemoglobin-positive HEPs are smaller with brown cytoplasm (18) (Fig. 1) (29). Consistent with our hypothesis, at a late phase of proliferation (culture day ∼14), cytospin preparations from control cultures (n = 11) showed a substantial number of cells with differentiated morphology, marked by smaller and dark-stained cells. In contrast, preparations from RTH*α* patients (n = 11) showed predominantly large light-stained cells, indicating that most cells had not yet entered the differentiation program [Fig. 2(a)].

The difference in cell size was confirmed by flow cytometry analysis. To distinguish large cells from small cells, a cutoff of ≥100 K was used, measured with forward scatter. The forward scatter showed that cells from RTH*α* patients (n = 11) had a larger mean size compared with cells of controls (n = 11; 41.7% of cells in patients vs 23.6% in controls had a forward scatter index larger than 100 K, *P* = 0.001), indicating that many RTH*α* HEPs were still expanding, whereas the majority of control HEPs had started their differentiation program [Fig. 2(b)].

HEPs express stage-specific cell surface proteins during different phases of erythropoiesis. CD-117 (c-Kit) is a marker of immature erythroblasts, and CD71 (transferrin receptor) expression increases during early differentiation, whereas GPA (CD235a) is a marker for mature erythroid cells (29, 32). During the later stages of proliferation (culture day ∼14), control HEPs (n = 11) were predominantly negative for c-Kit, as illustrated by an accumulation of HEPs in the c-Kit^negative^ subgate [Fig. 2(c)]. However, RTH*α* HEPs (n = 11) accumulated less in the c-Kit^negative^ subgate (37.2% in patients vs 74.5% in controls, *P <* 0.0001), corresponding to a delayed maturation stage. In addition, RTH*α* patients (n = 9, due to insufficient cells in two samples for GPA staining) showed less HEPs in the GPA^high^ subgate compared with controls (n = 9; 29.6% in patients vs 59.7% in controls, *P* = 0.003), again indicating that RTH*α* HEPs display a reduced or delayed spontaneous differentiation rate compared with control HEPs [Fig. 2(d)].

**Figure 3.**
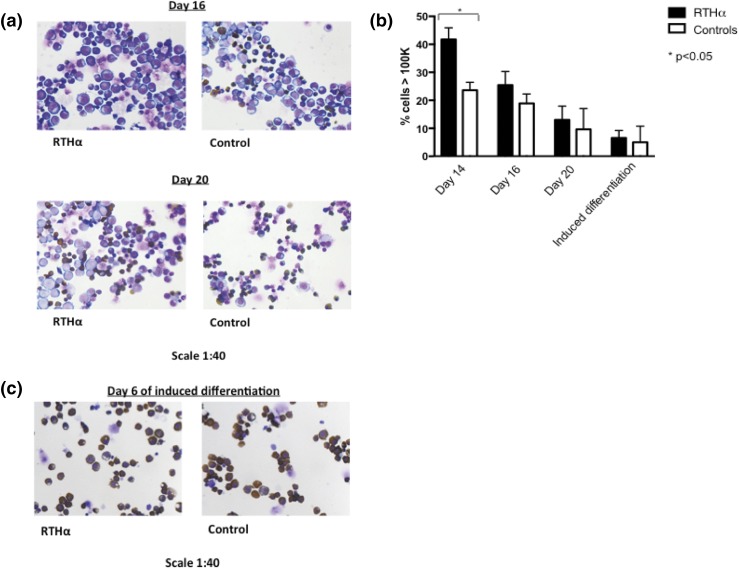
Differences between differentiating RTH*α*-derived and control HEPs decrease wherein RTH*α*-derived HEPs remain just behind control HEPs. (a) Cytospin preparations of RTH*α*-derived (P5; A263S) and control HEPs at day 16 (early spontaneous differentiation) and day 20 (late spontaneous differentiation). (b) Differences in cell size between RTH*α*-derived and control cultures during the different stages of differentiation, measured by flow cytometry analysis using forward scatter. To distinguish large cells from small cells, a cutoff of ≥100 K was used. (c) Cytospin preparations of RTH*α*-derived (P1; F397fs406X) and control HEPs after 6 days of induced differentiation, in which HEPs were cultured in StemSpan medium containing a high concentration of Epo, human serum, and transferrin.

Despite similar culture conditions, the period in which cells could be maintained in culture varied slightly between different experiments. Nevertheless, for all experiments, the longer the cells were cultured, the more cells differentiated fully and then became apoptotic, reducing availability of cell samples for experiments focusing on later stages of maturation. No differences in the extent of apoptosis between patient and control HEPs were observed. Patient cells (n = 7) and control samples (n = 5) were further monitored during culture days ∼15 to 20, still under proliferative conditions. The first signs of differentiation of RTH*α* cells were observed at ∼day 16, ∼2 days later than control cells. At this time point, the majority of the cells was reduced in size, but did not stain for hemoglobin [Fig. 3(a), top panel]. After ∼20 days of culture, apoptosis occurred in fully differentiated cells from most controls and RTH*α* patients, with no differences between cells with either frameshift/stop or missense mutations. Longer-term observations were made with the samples that still contained viable cells. After ∼20 days, the culture of control cells (n = 2) was terminated, as these cells were fully differentiated and started to disintegrate. In contrast, most RTH*α* cultures (n = 3) contained two viable subpopulations: differentiated, hemoglobin-positive cells, and immature blasts [Fig. 3(a), lower panel]. Although the number/percentage of blasts varied per patient/mutation, these immature cells could be maintained for an additional 5 to 7 days, followed by apoptosis. The differences in cell size between RTH*α* and control cultures during the different stages are shown in Fig. 3(b).

The delay in spontaneous differentiation of RTH*α* cells was also confirmed by FACS analysis. During differentiation, a large proportion of RTH*α* HEPs became negative for c-Kit and simultaneously increased the expression of GPA. Values for c-Kit and GPA expression in RTH*α*-derived cells reached levels comparable to controls, but with a delay of 4 days (Supplemental Fig. 2). During this period, expression values of the different cell surface markers in the control cells did not change, as maximal levels were already reached.

Taken together, these results show that onset of spontaneous differentiation is delayed in RTH*α* HEPs. However, when RTH*α* cells finally differentiate, differences between RTH*α* and control cells diminish, albeit with RTH*α* HEPs lagging behind control HEPs.

### Induced differentiation proceeds similarly in RTH*α* and control HEPs

Optimal and synchronous differentiation of HEP cells can be induced by changing the culture conditions. To study optimal or forced differentiation, HEPs were switched to StemSpan medium containing a high concentration of Epo (10 U/mL), human serum, and transferrin, whereas the proliferation factors (SCF and Dex) were removed. After 6 days of induced differentiation, cell populations of the remaining RTH*α* patients (n = 3) and controls (n = 2) predominantly consisted of small, dark-stained, and enucleated cells [Fig. 3(c)]. The expression of the different cell surface markers did not differ significantly between RTH*α* HEPs and control HEPs (Supplemental Fig. 2).

In summary, under forced differentiation conditions, RTH*α* cells differentiate nearly as well as control cells.

### T3 enhances spontaneous differentiation of RTH*α* HEPs

**Figure 4.**
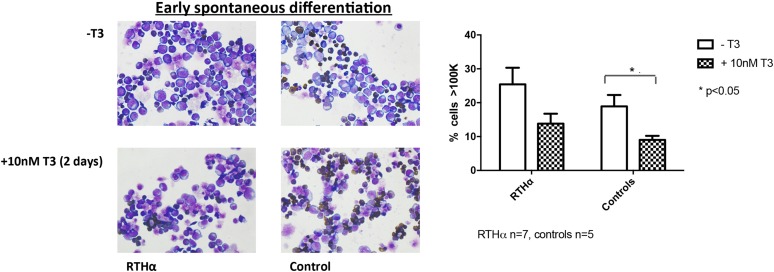
Cytospin preparations of RTH*α*-derived (P4; C392X, P5; A263S) and control HEPs at day 16 cultured with and without 10 nM T3 for 2 days. Addition of T3 resulted in an increased proportion of morphologically differentiated HEPs in both RTH*α* patients and controls. The decrease in cell size was significant in control HEPs.

It has been shown that addition of T3 does improve the differentiation characteristics of erythroid progenitors when grown *ex vivo* (16). To investigate the effect of T3 on RTH*α* HEPs, we exposed part of the RTH*α* (n = 7) and control HEP cultures (n = 5), at later stages of proliferation (∼day 14), to 10 nM T3. After 2 days, this resulted in an increased proportion of differentiated HEPs when compared with HEPs cultured without T3, with a similar effect in RTH*α* and control samples. Cells in treated samples were smaller (Fig. 4), stained less for c-Kit, and were more positive for GPA (Supplemental Fig. 3).

## Discussion

In the current study, we show that inactivating mutations in TR*α* affect the balance between proliferation and differentiation in HEPs. *Ex vivo* studies show that RTH*α* HEPs have a reduced capacity to differentiate spontaneously in comparison with control HEPs.

Several studies in humans have documented an association between hypothyroidism and reduced red blood cell counts, identifying mild anemia in 20% to 60% of hypothyroid patients (9, 33). Studies in avian and murine models point toward involvement of TR*α* in later stages of erythropoiesis (17, 18, 34–37). However, it is important to point out that there are significant differences in the physiology of normal erythropoiesis between murine and human contexts. For example, in mice, stress-induced erythropoiesis occurs in spleen, which is not the case in humans (18).

Because (mild) anemia, mostly normocytic normochromic, is also a common characteristic in RTH*α* patients (20–26, 28), we decided to investigate whether a defect in TR*α* affects the red cell maturation in RTH*α* patients. In this context, we sought to study whether tissue-specific hypothyroidism due to mutations in TRs affects erythropoiesis to a similar extent as primary hypothyroidism. The use of a well-validated cell culture protocol (29, 30) allowed us to expand the limited number of hematopoietic progenitors present in peripheral blood samples of both RTH*α* patients and healthy controls.

We were able to synchronize the development of HEPs at the proerythroblast stage, when cells show the first morphologically recognizable features of erythroid cells. From this stage, we studied the maturation of the HEPs using serum-free medium containing proliferation factors. Remarkably, RTH*α* HEPs continued to proliferate for a prolonged period, whereas control HEPs started to differentiate spontaneously from day 14. This delay in maturation and development was evident from both morphological appearance and expression of cell surface markers at this stage (2, 29). The serum-free culture medium used in our experiments contained low levels of T3 (∼0.5 nM), which may have been sufficient to enhance differentiation in control HEPs by activation of wild-type TR*α*. However, in RTH*α*-derived HEPs, such low T3 concentrations were probably unable to induce differentiation to a similar extent as these cells harbored mutant TR*α* with reduced T3 affinity, resulting in a prolonged proliferation phase (13).

For efficient terminal maturation, proliferation factors in the medium need to be replaced by differentiation factors (higher concentration of Epo, transferrin, serum). Addition of serum is necessary for complete downregulation of c-Kit and a further induction of GPA in human erythroblasts, both characteristics of terminal differentiation (30). Under such optimal differentiation conditions, differences between RTH*α*-derived and control HEPs decreased. This suggests that TR*α* mutations most likely affect the timing of onset of differentiation and influence the actual differentiation process itself to a lesser extent.

To investigate whether higher concentrations of T3 could overcome such delayed maturation dynamics, cultured HEPs during late proliferation were exposed to 10 nM T3. After 2 days, HEPs of both RTH*α*-derived and control T3-treated cells showed a modest decrease in cell size, indicating an increased proportion of spontaneous differentiation. This observation is consistent with previous findings from Leberbauer *et al.* (30), showing that proliferating HEPs treated with T3 have a gradual decrease in cell size, reduced proliferation rates, and higher hemoglobin content.

Previous studies indicate that exposure of normal HEPs to T3 does not affect expression of differentiation cell surface markers (30), and we confirmed these findings with control HEPs in this study. In contrast, RTH*α* HEPs exposed to 10 nM T3 showed a slight increase in CD71 and GPA expression together with downregulation of c-Kit levels. One explanation for these findings is that levels of T3 present in normal medium are sufficient to permit differentiation of control HEPs with higher exogenous T3 concentrations following addition of exogenous hormone only shifting the balance of cells achieving terminal maturation. In contrast, in RTH*α*-derived HEPs, harboring dysfunctional TR*α*, higher T3 concentrations could help overcome the TR*α* defect, thereby accelerating the onset of spontaneous differentiation. Previous characterization (20–28) of TR*α* mutations identified in RTH*α* cases indicates variable dysfunction with frame shift and nonsense mutations being markedly more deleterious than missense mutations (with some proximal receptor mutations also affecting TR*α*2). In this context, both in the current study and in reports of other published RTH*α* cases, there is no association between the degree of anemia and the severity of mutations in TR*α*. Furthermore, there is no relationship between hemoglobin concentrations or red blood cell number and TH levels in cases, all showing inherent variation among different subjects. Given these *in vivo* findings in RTH*α*, it is not surprising that the maturation dynamics of RTH*α* HEPs, harboring TR*α* mutations of varying severity, did not differ. Despite the anemia, the number of reticulocytes was not increased in RTH*α* patients, suggesting that the release of immature erythrocytes into the circulation is not altered. In addition, no major abnormalities in other blood cell lineages were detected. However, we were not able to measure the life span of erythrocytes in our patients *in vivo*; accordingly, we cannot discount the possibility that the life span of RTH*α* erythrocytes is altered compared with healthy controls.

Cultured, RTH*α*-derived HEPs responded to 10 nM T3 exposure, whereas LT4 treatment did not correct anemia in most patients with RTH*α*. One possible explanation for this discrepancy is that mutant TR*α* affects earlier phases of erythrocyte development in the bone marrow in RTH*α* patients, which we have not been able to examine in this study. In the current project, we studied relatively late stages of erythrocyte maturation, in which T3 is known to favor differentiation of erythrocytes (38). In this phase, addition of higher T3 concentrations stimulated the onset of differentiation in both RTH*α*-derived and control HEPs. However, it remains unclear whether T3 via TR*α* similarly affects earlier stages of erythrocyte development and, if so, whether LT4 treatment of RTH*α* patients can rescue mutant TR*α* function in this earlier phase.

In addition, we cannot discount the possibility that culture of isolated HEPs *ex vivo* enables different facets of the maturation process to be studied and regulated by exposure to T3, whereas it is more difficult to influence the likely complex interactions between HEPs with other cell types and factors that control the maturation process *in vivo*, with TH treatment. Thus, in addition to intrinsic differences between RTH*α* and normal erythrocyte progenitors, it is conceivable that such added interactions contribute to the anemia of RTH*α* patients.

In conclusion, by studying erythroid progenitor cells from RTH*α* patients *ex vivo*, we have shown that both TH and TR*α* play a role in the later phases of human erythropoiesis. Erythrocyte progenitors from RTH*α* patients exhibit defective maturation capacity, with later onset of, and a slower progression through, the terminal differentiation program.
