# Supplementary figures and images for: Anemia in Patients With Resistance to Thyroid Hormone α: A Role for Thyroid Hormone Receptor α in Human Erythropoiesis
Source: J Clin Endocrinol Metab. 2017 Jul 11;102(9):3517–25. doi: 10.1210/jc.2017-00840 (PMC5587074; doi:10.1210/jc.2017-00840)

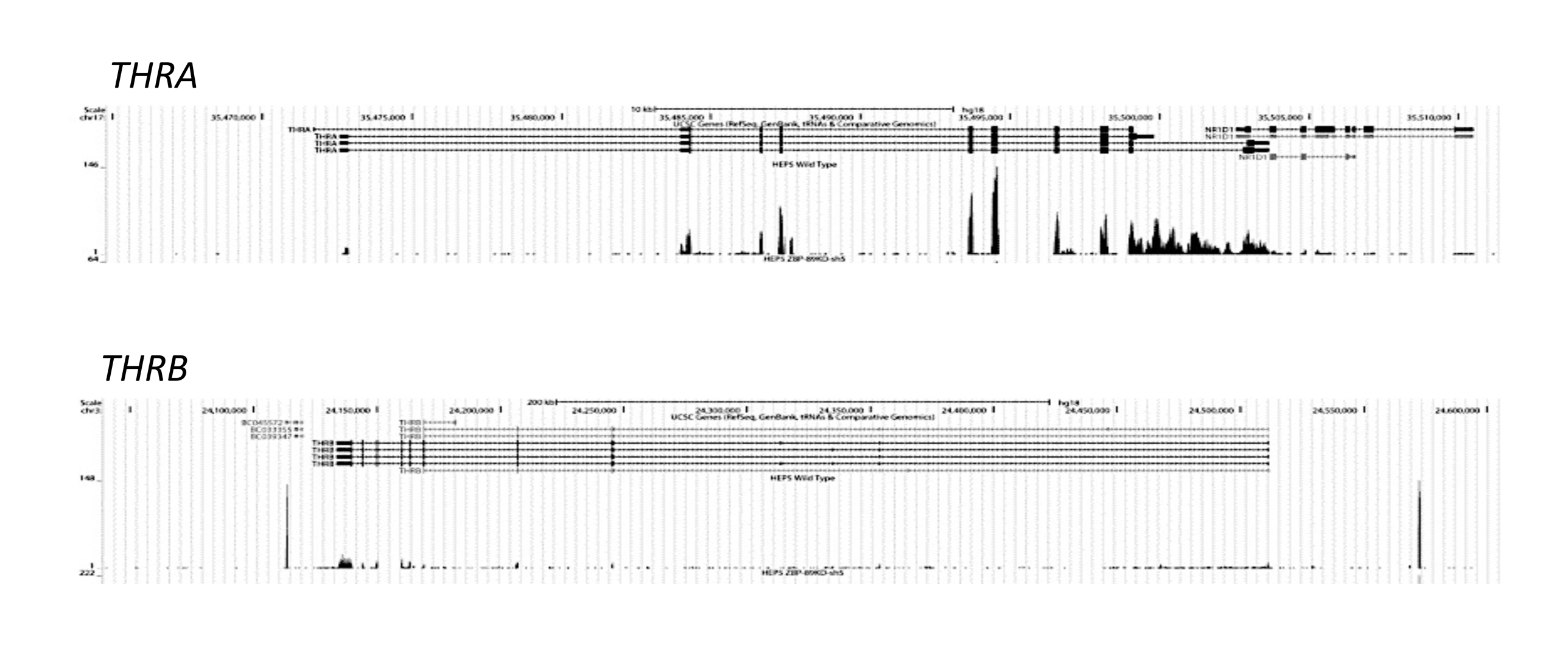

Supplement: Supplementary file 1 [file jc.2017-00840.sf1.tif]

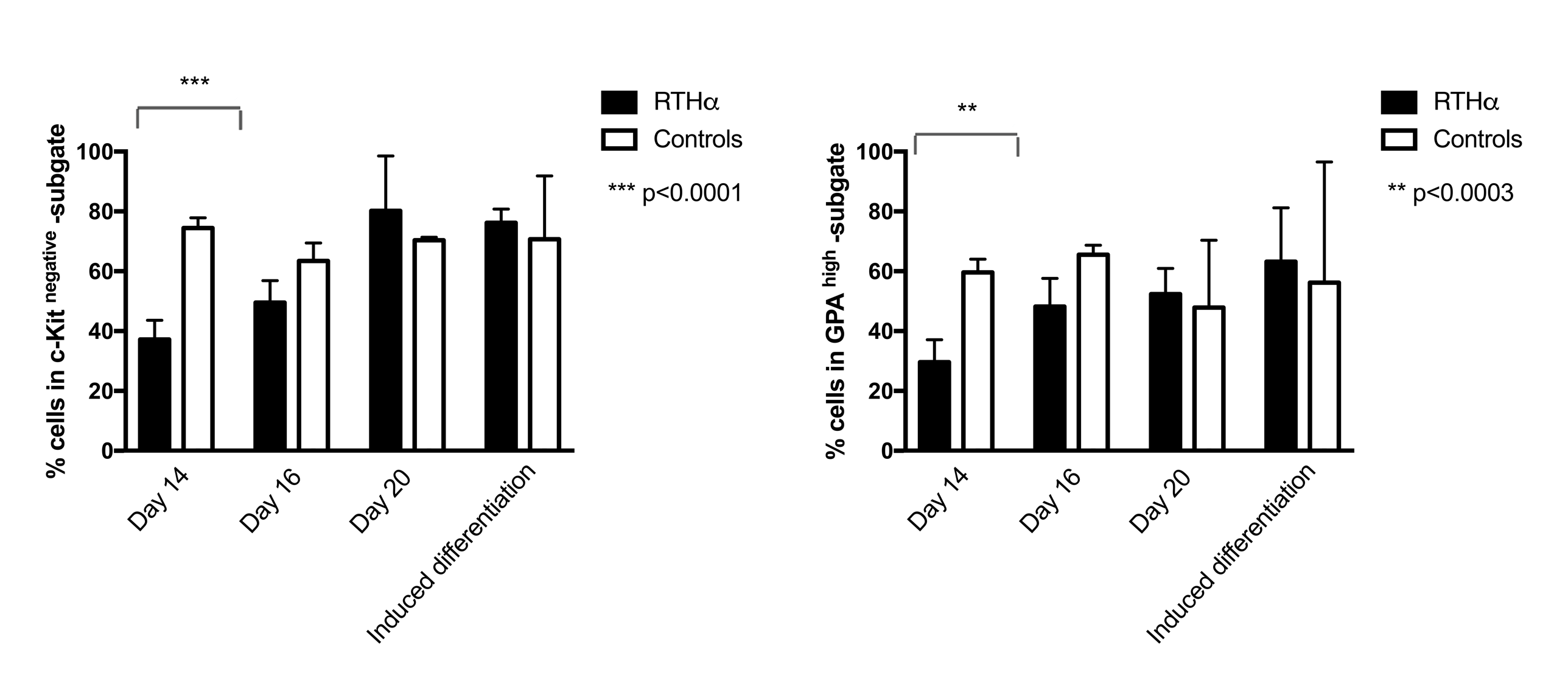

Supplement: Supplementary file 2 [file jc.2017-00840.sf2.tif]

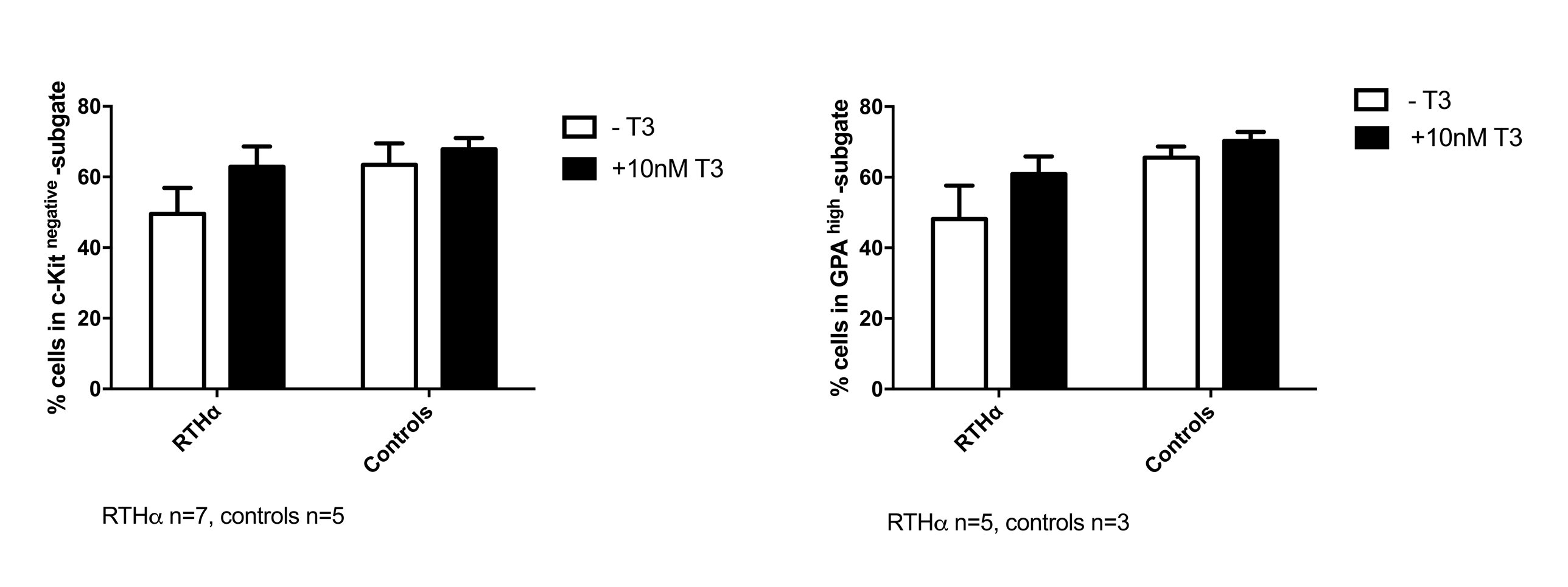

Supplement: Supplementary file 3 [file jc.2017-00840.sf3.tif]
